# Modified Vaccinia Virus Ankara Can Induce Optimal CD8^+^ T Cell Responses to Directly Primed Antigens Depending on Vaccine Design

**DOI:** 10.1128/JVI.01154-19

**Published:** 2019-10-15

**Authors:** Yik Chun Wong, Sarah Croft, Stewart A. Smith, Leon C. W. Lin, Tania Cukalac, Nicole L. La Gruta, Ingo Drexler, David C. Tscharke

**Affiliations:** a John Curtin School of Medical Research, The Australian National University, Canberra, Australian Capital Territory, Australia; b Department of Microbiology and Immunology, University of Melbourne, The Peter Doherty Institute for Infection and Immunity, Melbourne, Victoria, Australia; c Monash Biomedicine Discovery Institute and Department of Biochemistry and Molecular Biology, Monash University, Clayton, Victoria, Australia; d Institute for Virology, Düsseldorf University Hospital, Heinrich Heine University, Düsseldorf, Germany; University of Illinois at Urbana Champaign

**Keywords:** CD8^+^ T cells, CTL, cytotoxic T cells, MVA, modified vaccinia virus Ankara, antigen presentation, antigen processing, live vector vaccines, vaccinia virus

## Abstract

A variety of strains of vaccinia virus (VACV) have been used as recombinant vaccine vectors with the aim of inducing robust CD8^+^ T cell immunity. While much of the pioneering work was done with virulent strains, such as Western Reserve (WR), attenuated strains such as modified vaccinia virus Ankara (MVA) are more realistic vectors for clinical use. To unify this literature, side-by-side comparisons of virus strains are required. Here, we compare the form of antigen that supports optimal CD8^+^ T cell responses for VACV strains WR and MVA using equivalent constructs. We found that for multiple antigens, minimal antigenic constructs (epitope minigenes) that prime CD8^+^ T cells via the direct presentation pathway elicited optimal responses from both vectors, which was surprising because this finding contradicts the prevailing view in the literature for MVA. We then went on to explore the discrepancy between current and published data for MVA, finding evidence that the expression locus and in some cases the presence of the viral thymidine kinase may influence the ability of this strain to prime optimal responses from antigens that require direct presentation. This extends our knowledge of the design parameters for VACV vectored vaccines, especially those based on MVA.

**IMPORTANCE** Recombinant vaccines based on vaccinia virus and particularly attenuated strains such as MVA are in human clinical trials, but due to the complexity of these large vectors much remains to be understood about the design parameters that alter their immunogenicity. Previous work had found that MVA vectors should be designed to express stable protein in order to induce robust immunity by CD8^+^ (cytotoxic) T cells. Here, we found that the primacy of stable antigen is not generalizable to all designs of MVA and may depend where a foreign antigen is inserted into the MVA genome. This unexpected finding suggests that there is an interaction between genome location and the best form of antigen for optimal T cell priming in MVA and thus possibly other vaccine vectors. It also highlights that our understanding of antigen presentation by even the best studied of vaccine vectors remains incomplete.

## Abstract (precis)

Recombinant vaccines based on vaccinia virus and particularly attenuated strains such as MVA are in human clinical trials, but due to the complexity of these large vectors much remains to be understood about the design parameters that alter their immunogenicity. Previous work had found that MVA vectors should be designed to express stable protein in order to induce robust immunity by CD8^+^ (cytotoxic) T cells. Here, we found that the primacy of stable antigen is not generalizable to all designs of MVA and may depend where a foreign antigen is inserted into the MVA genome. This unexpected finding suggests that there is an interaction between genome location and the best form of antigen for optimal T cell priming in MVA and thus possibly other vaccine vectors. It also highlights that our understanding of antigen presentation by even the best studied of vaccine vectors remains incomplete.

## INTRODUCTION

Vaccinia virus (VACV) was one of the first vectors for recombinant vaccines and candidates have now progressed to clinical trials. The generation of strong CD8^+^ T cell immunity to foreign antigens encoded in a VACV vector is well agreed upon, in mouse (1–3), nonhuman primate (4–6), and human (7, 8) models. However, while the bulk of historic studies aimed to understand antigen presentation from VACV were done with virulent strains such as Western Reserve (WR), these viruses have an unacceptable risk profile and are not suitable for use as human vaccines. Safer alternatives have emerged and among these is modified vaccinia virus Ankara (MVA); however, there is a lack of studies that examine MVA and WR in parallel, and such studies are required to unify a broad literature.

MVA is a hyperattenuated VACV strain that lacks immune evasion proteins and virulence factors and does not productively replicate in human tissue (9–11). Clinical trials with MVA as a smallpox vaccine have demonstrated safety, even in HIV- positive individuals (12, 13). These characteristics, along with the capacity to insert up to 25 kbp of foreign genes (10, 14), makes MVA an ideal vector for recombinant vaccines, a potential that is being pursued through the development pipeline into clinical trials (15–24). Further work is progressing to improve immunogenicity and assess the risks of broad release of MVA as a recombinant vaccine (25, 26), but these are large vectors, leaving many aspects of their biology incompletely studied.

A key design parameter for vaccines that induce CD8^+^ T cells is the form of antigen expressed, be that full-length protein and other stable polypeptides or, conversely, rapidly degraded antigen forms, such as ubiquitin-antigen fusion proteins, artificial polyepitopes, and minimal epitope constructs (the latter are referred to here as minigenes). This is an important consideration because of the manner in which each of these is processed and presented on major histocompatibility complex (MHC) class I to activate or prime CD8^+^ T cells (27). Epitope minigenes bypass the requirement for proteasomal processing from larger polypeptides and so enter the antigen presentation pathway very efficiently in infected cells and are therefore present in great abundance on MHC. However, these short peptides are rapidly degraded, being shown to have a half-life of under 10 s (28). For this reason, although minigenes, and indeed all rapidly degraded polypeptides, prime well by the direct presentation pathway, i.e., via vaccine-infected dendritic cells (DCs), they do not survive long enough to be picked up and cross presented by uninfected DCs (29–31). Epitopes from stable antigens are directly presented on virus-infected DCs at various levels, but in many cases they can also be cross primed (27). Direct presentation is linked closely with translation, and so the structures or functions of full-length proteins are not associated with the levels of presentation by this pathway, unlike factors such as the efficiency of processing and the rate of translation (27, 32–35). Cross presentation is more likely to be influenced by the features of proteins, but with the exception of stability this has not been systematically explored. Further, the relative roles of these two pathways remain difficult to dissect for stable proteins, so it is safest to assume that both pathways may be used; but if neither is efficient, the responses elicited may be modest (36, 37). Finally, we note that while epitope minigenes are themselves not realistic vaccine constructs, because they prime responses to a single epitope presented by a single MHC they are a good model for all rapidly degraded antigen forms, as noted above.

Many studies over 20 years have shown that minigenes (or rapidly degraded polypeptides) encoded by VACV WR induce a CD8^+^ T cell immune response that is always as strong and often significantly stronger than a corresponding full-length stable antigen (37–41). Importantly, this finding holds irrespective of the nature or function of the full-length protein and implies that for recombinant VACV strain WR (rWR), optimizing antigens for effective direct presentation is always adequate and often ideal. In contrast to this, for MVA the prevailing view in the field is that minigenes and other rapidly degraded polypeptides prime poorly. This is based on a key study that directly examined priming requisites for this vector and concluded that stable antigens are best because cross priming is dominant (42). Notably, that report also examined multiple antigens, always finding the same result, suggesting that the finding would generalize broadly. This has been more recently supported by evidence that a second wave of presentation of antigens, which for MVA occurs exclusively by cross presentation, is required for CD4^+^ T cell help and full development of CD8^+^ T cell responses (43). Further, mouse knockout models have found that molecules required for cross presentation impact the immunogenicity of MVA more than WR (44). However, many studies have found that MVA can infect DCs, and initial activation of CD8^+^ T cells by direct priming has been visualized (42, 43, 45–48). In addition, effective priming of a minigene by MVA has been noted as an incidental finding elsewhere (37).

The purpose of this study was to reconcile these opposing findings for WR and MVA, as well as the possible discrepancy across findings with MVA. To do this, we revisited the preferred form of antigen for CD8^+^ T cell priming by recombinant VACV (rVACV) based on strains WR and MVA. Our approach at the outset was to examine a broad range of antigens, which included: herpes simplex virus (HSV) glycoprotein B (gB), a highly immunodominant viral glycoprotein; influenza virus PB1F2, a very weakly immunogenic intracellular viral protein; VACV B8, a dominant native antigen of VACV; and ovalbumin, a classic model antigen for which published data already exist for WR and MVA. By choosing this broad array of antigen/epitope pairs, we sought to identify unifying patterns.

## RESULTS

### A minimal epitope construct of HSV gB_498_ is more immunogenic than the full-length protein when expressed both from VACV WR and from MVA.

The first foreign antigen/epitope we examined was gB of HSV, from which the highly immunodominant epitope gB_498_ is derived (49). Recombinant VACV strain WR (rWR) viruses expressing full-length gB and an endoplasmic reticulum (ER)-targeted minigene (minigene-gB_498_) from the *J2R* gene under the p7.5 promoter have been published previously (50, 51). ER-targeted epitope minigenes deliver minimal epitope sequences directly into the ER, and so their presentation in infected cells is generally independent of the transporters associated with antigen presentation, but they behave similarly to cytosolic epitope minigenes and other rapidly degraded antigen forms in terms of priming pathway preference (30, 52). *J2R* encodes the thymidine kinase (TK), and this function is lost in viruses made in this way due to insertional inactivation. This insertion site is referred to here as the TK locus. For this study, we generated recombinant MVA (rMVA) viruses that were matched to the rWRs above in the forms of antigen, site of insertion, and promoter. As controls, we used viruses that had insertions in the TK locus but that expressed no foreign viral protein.

**FIG 1:**
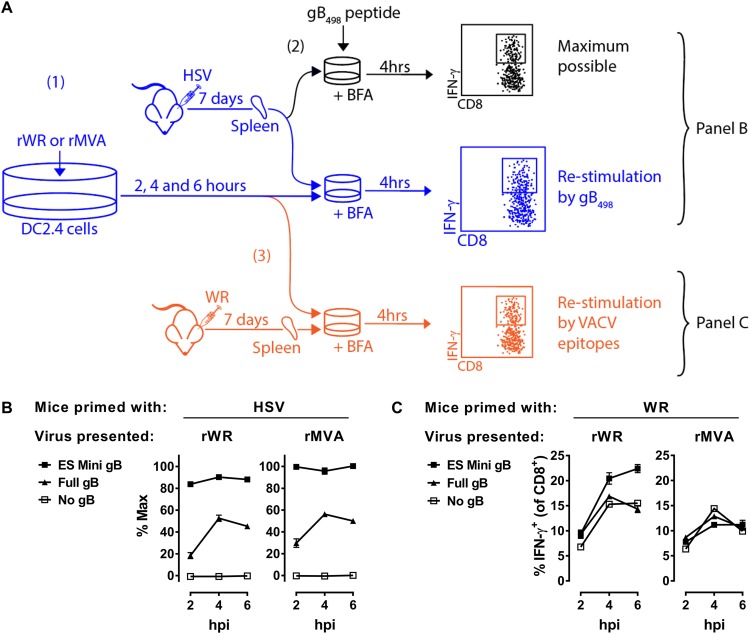
Full-length gB and minigene-gB_498_ are expressed from rVACVs. (A) Diagram of experimental plan. DC2.4 cells were infected with rVACVs for 2, 4, or 6 h, and then the levels of HSV gB_498_ presentation on MHC-I were determined by coculture with HSV-immune splenocytes in the presence of brefeldin A, followed by flow cytometry to identify IFN-γ^+^ CD8^+^ T cells (blue, step 1). The HSV-immune splenocytes were separately stimulated with gB_498_ peptide for 4 h, and the percentages of CD8^+^ T cells that were IFN-γ^+^ were measured to provide a maximum possible gB_498_-specific response (black, step 2). Results from cocultures of infected cells with HSV-immune splenocytes are shown as a percentage of this maximum possible response. Finally, separate aliquots of the same infected cultures were incubated with VACV-immune splenocytes, and IFN-γ^+^ CD8^+^ T cells were again counted to determine the general levels of infection and antigen presentation (orange, step 3). (B) Data reflecting the levels of gB_489_ presented on triplicate cultures of rWR- or rMVA-infected cells from the experiment described above. The virus strain is shown above graphs, and the form of gB antigen expressed is in the key. (C) Data reflecting levels of VACV antigen presentation on the same triplicate cultures of rWR- and rMVA-infected cells. Means and standard errors of triplicates are shown; some errors are obscured by data points. The experiment was repeated with similar results.

One of the limitations of using epitope minigenes is that their expression cannot be detected by conventional methods, such as Western blotting. For this reason, we needed a way to detect the epitopes presented in association with MHC class I (MHC-I) on cells infected with these viruses to ensure that all were being expressed. Further, by detecting the level of presentation, we have some indication of how well each of the viruses might perform in direct priming, assuming that the vectors can infect the relevant DCs *in vivo*. To do this for HSV gB_498_, we used an assay based on the ability of cells infected with our rVACV *in vitro* to restimulate CD8^+^ T cells from mice acutely infected with HSV (Fig. 1A). We used the C57BL/6-derived cell line DC2.4, and after 2, 4, or 6 h of infection with the rVACVs, cocultured these cells with splenocytes taken from a mouse 7 days after infection with HSV. The coculture was done in the presence of brefeldin A and restimulation of CD8^+^ T cells was determined by the detection of intracellular gamma interferon (IFN-γ) by flow cytometry (Fig. 1A depicted in blue). We cultured another aliquot of the same splenocytes with 1 × 10^−7^ M gB_498_ peptide and again measured the intracellular IFN-γ to establish a maximum possible response (Fig. 1A, depicted in black). This allowed the response from rVACV-infected cells to be plotted as a percentage of the maximum possible response. This was necessary for standardization across experiments. As expected, for both rWR and rMVA, cells infected with viruses expressing minigene-gB_498_ were the better at restimulating CD8^+^ T cells from HSV-infected mice than those infected with the viruses expressing full-length gB (Fig. 1B). At the same time, viruses with no form of gB failed to restimulate the HSV-immune splenocytes, showing there was no cross-reactivity between HSV- and VACV-specific CD8^+^ T cells. Finally, to ensure that these results were not due to differing efficiencies of the infections or other factors that might impact antigen presentation in general across the various viruses, portions of the same batches of infected cells used above were tested for their ability to stimulate CD8^+^ T cells from VACV WR-infected mice (Fig. 1A, depicted in orange). Restimulation of WR-immune CD8^+^ T cells was similar among the cultures infected with the three rWRs and also across those infected with the three rMVA (Fig. 1C). These controls suggest that the infections were all similarly efficient, at least within a VACV strain. Taken all together, we interpret the results of these experiments as showing that (i) full-length gB and minigene-gB_498_ were expressed and presented on MHC-I as anticipated from the rWR and rMVAs but (ii) that minigene-gB_498_ was a more efficient form of antigen in terms of direct presentation of the gB_498_ epitope on infected cells.

**FIG 2:**
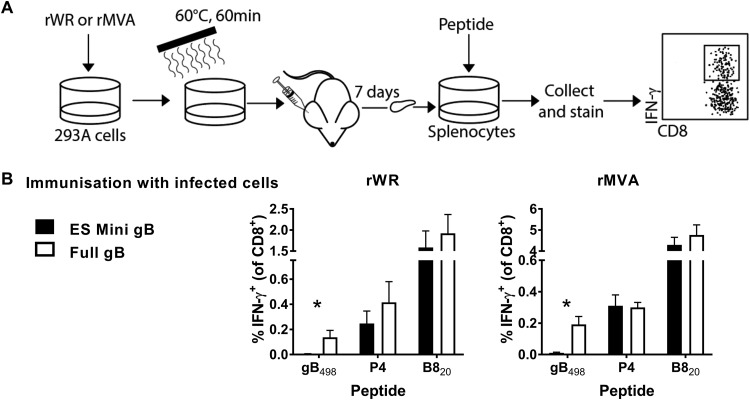
Cells infected with rVACVs expressing full-length gB, but not minigene-gB_498_, can cross prime CD8^+^ T cells *in vivo*. (A) Diagram of the experimental plan. Human 293A cells were infected with rWR or rMVA for a total of 6 h, and then counted and heat treated to eliminate any infectivity. Mice were immunized with these cells by i.d. injection and, after 7 days, epitope-specific CD8^+^ T cell responses were measured by *in vitro* culture with peptides and flow cytometry for CD8 and IFN-γ. (B) Data from the experiment described in panel A. Mice were immunized with cells infected with the VACV strain as shown above the graphs expressing the form of gB_498_ as shown in the key. The epitopes are shown on the *x* axis; P4 is the sum of responses to A47_138_, A47_171_, L2_53_, and A3_270_. Means and standard errors of data from six mice combined from two independent experiments are shown (*, *P* < 0.05).

Next, we used an established vaccination method to determine whether the gB-containing antigens expressed by our viruses in infected cells might be able to cross prime CD8^+^ T cells *in vivo* (30, 37, 52, 53). As a source of antigen, human 293A cells were infected with rWR and rMVA viruses and then heat killed to destroy any infectivity, which includes residual viral inoculum as well as any replicated virus. These cells were used to vaccinate mice by intradermal (i.d.) injection of ear pinnae and, after 7 days, epitope-specific responses were determined by a short *ex vivo* culture with synthetic peptides and staining for CD8 and IFN-γ (Fig. 2A). We measured responses to gB_498_ and, to ensure similar immunization across viruses, selected VACV epitopes, including the dominant B8_20_ and a set of less-dominant epitopes comprising A47_138_, A47_171_, L2_53_, and A3_270_, for which responses were summed and are referred to as P4. All of these epitopes are shared between WR and MVA (54, 55). Both for rWR and for rMVA, only cells infected with viruses that expressed the full-length gB were able to prime a gB_498_-specific CD8^+^ T cell response above background in mice (Fig. 2B). The sizes of these responses were low, at around 0.2% of CD8^+^ T cells, but this is in the range published for similar types of experiments (30, 37). At the same time, rWR-infected cells primed responses to the VACV epitopes to similar levels irrespective of the form of gB expressed, and the same was observed for the pair of rMVAs. These data suggest that full-length gB but not minigene-gB_498_ can be cross primed *in vivo*. We do not interpret this result to mean that the full-length antigens are necessarily cross primed after an infection of mice with these viruses, just that to the limit of detection of this assay the minigenes are not able to be cross presented, consistent with published results for other antigens (30, 56).

**FIG 3:**
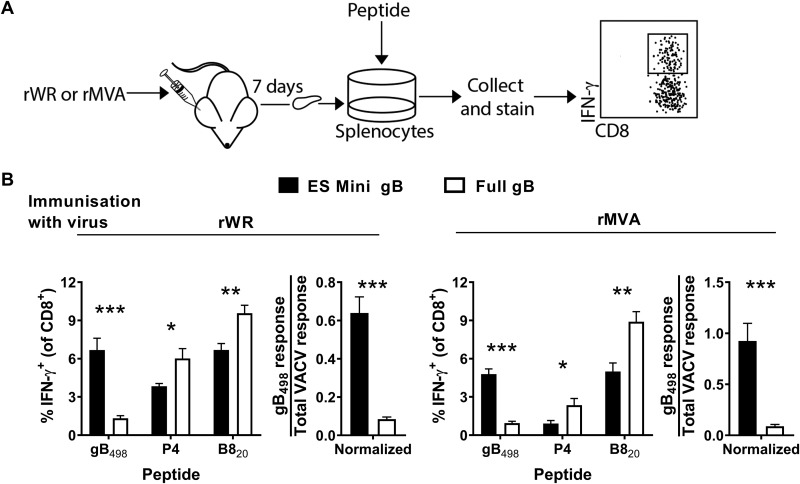
Minigene-gB_498_ is more effective than full-length gB for priming CD8^+^ T cells when expressed from rWR and rMVA. (A) Diagram of the experimental plan. Mice were infected with rWR and rMVA viruses expressing versions of HSV gB_498_ by i.d. injection and, after 7 days, epitope-specific CD8^+^ T cell responses were measured by *in vitro* culture with peptides and flow cytometry for CD8 and IFN-γ. (B) Data from the experiment described in panel A. Mice were immunized with rWRs (left) or rMVAs (right) expressing the form of gB_498_ as shown in the key. The peptides are shown on the *x* axis. The graph on the right for each VACV strain shows the gB_498_-specific response divided by the total VACV-specific response (i.e., the sum of P4 and B8_20_) to account for any differences in infection across the viruses. Means and standard errors of data from six mice combined from two independent experiments are shown (*, *P* < 0.05; **, *P* < 0.01; ***, *P* < 0.001).

Finally, we wanted to determine for rWR and rMVA the form of HSV gB that was most immunogenic after a standard infection of mice, which is the key experiment with these viruses. To do this, mice were infected by the i.d. route with the gB_498_-presenting rWR and rMVA viruses, and CD8^+^ T cell responses to gB_498_, P4, and B8_20_ in the spleen were measured 7 days later (Fig. 3A). As published previously for rWR (51), minigene-gB_498_ primed significantly more CD8^+^ T cells than full-length gB, but more surprisingly, this result was recapitulated by the rMVA viruses (Fig. 3B). Indeed, the strength of the minigene-gB_498_ priming from both vectors appeared to compete with native VACV epitopes, including the usually dominant B8_20_ epitope, such that responses were reduced in mice infected with the minigene, compared with full-length gB-expressing viruses. However, we cannot be absolutely sure that reduction of response to the VACV epitopes does not reflect some disadvantage in infection or general antigen presentation for viruses expressing minigene-gB_498_. For this reason, we also plotted gB_498_-specific responses normalized against the total VACV-specific response (sum of P4 and B8), which highlighted further the difference between minigene and full-length HSV gB-expressing viruses (Fig. 3B).

From these data, we concluded that for the highly dominant HSV gB_498_ epitope, the priming requisites for rWR and rMVA are the same when similar constructs are compared, the form of gB optimized for direct presentation (Fig. 1) and not able to cross prime (Fig. 2) being the most immunogenic (Fig. 3).

### Minigene-IAV PB1-F2_62_ is more immunogenic than full-length PB1-F2 when expressed from VACV strains WR and MVA.

We were concerned that the gB_498_ epitope might not be representative, perhaps because of its immunodominant nature or its structure as a surface glycoprotein, so we wanted to determine whether the results above would be consistent for a very weak, cytoplasmic antigen. We chose a subdominant influenza A virus (IAV) antigen/epitope, namely, the nonstructural PB1-F2 protein and its epitope PB1-F2_62_ (57). The viruses used were matched with those expressing HSV gB above in promoter and insertion site. The rWRs were made by others (53, 58), but the rMVAs were made for this study. The same TK^–^ control viruses were used.

**FIG 4:**
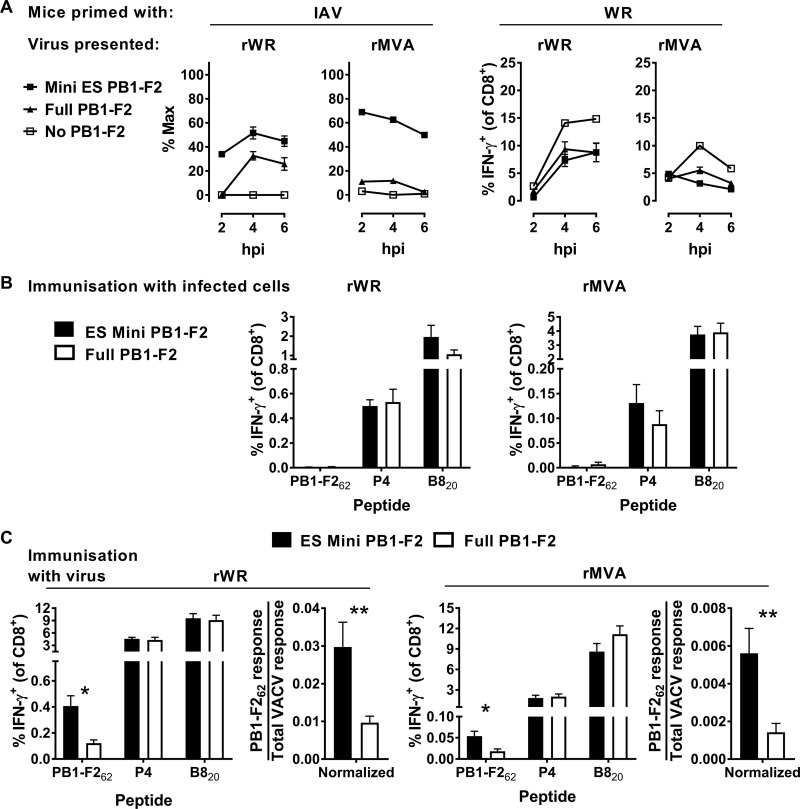
Presentation to, and priming of, CD8^+^ T cells by PB1-F2_62_ expressed in different forms by rWR and rMVA. (A) Results obtained according to the experimental design in Fig. 1A show the extent of restimulation of CD8^+^ T cells from IAV-immune (left) or VACV WR-immune (right) splenocytes by cells infected by the virus strain, as shown above graphs, expressing the forms of PB1-F2_62_ as shown in the key. For the cocultures with IAV-immune splenocytes (left), data are presented relative to the maximum possible value obtained by stimulation of the same spleen cells with PB1-F2_62_ peptide. Means and standard errors of triplicates are shown. The experiment was repeated with similar results. (B) Results obtained according to the experimental design shown in Fig. 2A, except viruses expressed versions of PB1-F2_62_ (as shown). Epitope-specific responses are shown as the percentage of CD8^+^ T cells making IFN-γ. (C) Results obtained according to the experimental design in Fig. 3A. Mice were infected with rWR and rMVA viruses expressing versions of PB1-F2_62_ (as shown) and, 7 days later, the epitope-specific responses were measured. The graph on the right for each VACV strain shows the PB1-F2_62_-specific response divided by the total VACV-specific response. For panels B and C, the means and standard errors of data from nine mice from three independent experiments are shown (*, *P* < 0.05; **, *P* < 0.01).

Following the experimental pattern established in the previous section, we first tested the ability of these rVACVs to express their antigens in infected cells, as reflected by the presentation of PB1-F2_62_ on MHC-I. The experimental scheme was as shown in Fig. 1A, but this time we used splenocytes from IAV-primed mice to detect the presentation of PB1-F2_62_. All viruses with PB1-F2, irrespective of the form, were detected by the IAV-immune CD8^+^ T cells, but the control WR and MVA were not detected. As expected, both for rWR and for rMVA, cells infected with viruses expressing minigene-PB1-F2_62_ were the best at restimulating CD8^+^ T cells from IAV-infected mice (Fig. 4A, left). The control restimulations of WR-immune splenocytes showed that the VACV-derived epitopes were presented equally across the set of rWRs and across the set of rMVAs, so that within each strain the infection was equally efficient (Fig. 4A, right). We interpret these results, taken together, to show that these sets of viruses expressed their antigens and that there was more efficient presentation of PB1-F2_62_ on infected cells expressing the minigene than the full-length PB1-F2.

Next, we used the *in vivo* assay based on immunization of mice with infected and heat-treated 293A cells to determine whether full-length and minigene-PB1-F2 were able to cross prime CD8^+^ T cells. As above, we measured responses against PB1-F2_62_ and as controls, VACV epitopes including B8_20_, and the P4 set of epitopes. None of the four viruses was able to prime a PB1-F2_62_-specific CD8^+^ T cell response that was clearly above background in these experiments, despite the VACV antigens being able to elicit responses at the expected levels (Fig. 4B). These data are consistent with the poor immunogenicity of PB1-F2_62_ when mice were immunized with IAV-infected MC57G cells lacking TAP (59). Further, it is possible that expression of PB1-F2 protein is not well tolerated by cells in the context of infection with one or both rVACVs, and this is reflected in the lack of a response. Arguing against this is that the responses to the native VACV epitopes (P4 and B8) were similar for the viruses expressing full-length PB1-F2 (presumably functional) and minigene-PB1-F2_62_ (not functional). So, for PB1-F2_62_ we were not able to confirm whether either form of antigen was able to cross prime CD8^+^ T cells, probably due to the poor immunogenicity of this epitope.

Finally, mice were infected with the PB1-F2_62_-presenting viruses to determine for each vector which form of antigen was most immunogenic in a live-virus vaccination. All responses to PB1-F2_62_ were exceptionally weak, being just above background; however, all the average responses were above zero by more than the 95% confidence level (not shown). Further, the minigene-PB1-F2_62_ construct primed significantly more CD8^+^ T cells than full-length PB1-F2, when expressed both from rWR and from rMVA (Fig. 4C). The CD8^+^ T cell response to the native VACV epitopes appeared to be similar across the viruses from each strain; however, to take this into account formally, we normalized PB1-F2_62_-specific responses against the total anti-VACV response. Doing this confirmed that the responses primed by minigenes were stronger. Therefore, we conclude that minigenes are the optimum form of PB1-F2_62_ for stimulating CD8^+^ T cell responses for rWR and rMVA vectors, even though responses were very low and close to the limit of detection.

With the exception of our inability to detect cross priming from infected cells, these data recapitulate what was seen for HSV gB_498_: there was no difference in the preferred form of antigen for priming CD8^+^ T cells between rWR and rMVA, and directly presented minigenes were more immunogenic than a full-length antigen.

### Minigene-B8_20_ is more immunogenic than full-length B8 when expressed from MVA.

The two antigens examined thus far were expressed from the same promoter and were inserted into the TK gene, so we then extended the study to a VACV antigen expressed by its own promoter, from its native location. We chose the highly immunogenic VACV B8_20_ epitope and compared antigen presentation and immunogenicity from wild-type viruses and from recombinants where the *B8R* open reading frame had been replaced with a short sequence encoding a B8_20_ minigene. It should be noted here that B8 from MVA is truncated and predicted to be nonfunctional, so the nonrecombinant WR and MVA viruses that serve to provide full-length B8 protein are not strictly equivalent. However, (i) in well-controlled studies WR B8 fails to function in mice due to an inability to adequately bind mouse IFN-γ, so neither WR or MVA form of this protein should impact immune responses (60), and (ii) our data from Fig. 2 and 4B, in which B8 is used as a control native VACV antigen, confirm that both forms of B8 are stable enough to support very effective cross priming.

**FIG 5:**
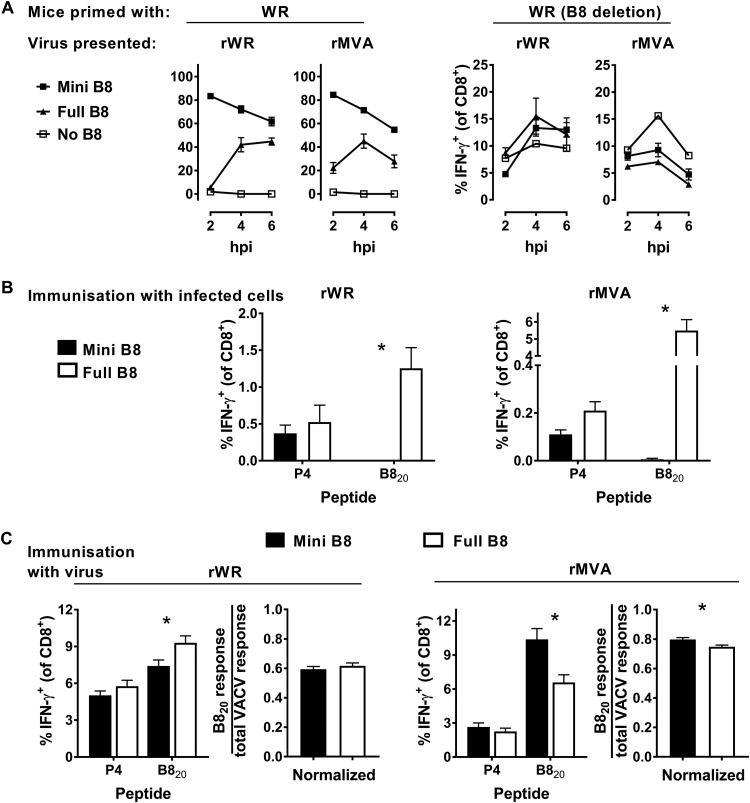
Presentation to, and priming of, CD8^+^ T cells by B8_20_ expressed in different forms by rWR and rMVA. (A) Results obtained according to the experimental design in Fig. 1A showing the extent of restimulation of CD8^+^ T cells from IAV-miniB8_20_-immune (left) or VACV WR(delB8)-immune (right) splenocytes by cells infected by the virus strain, as shown above the graphs, expressing the forms of B8_20_ as shown in the key. For the cocultures with IAV-miniB8_20_-immune splenocytes (left), the data are presented relative to the maximum possible value obtained by stimulation of the same spleen cells with B8_20_ peptide. Means and standard errors of triplicates are shown. The experiment was repeated with similar results. (B) Results obtained according to the experimental design shown in Fig. 2A, except the viruses expressed versions of B8 (as shown). Epitope-specific responses are shown as the percentage of CD8^+^ T cells making IFN-γ. (C) Results obtained according to the experimental design in Fig. 3A. Mice were infected with rWR and rMVA viruses expressing versions of B8 (as shown) and, 7 days later, the epitope-specific responses were measured. The graph on the right for each VACV strain shows the B8-specific response divided by the total VACV-specific response. For panels B and C, means and standard errors of data from 15 mice from five independent experiments are shown (*, *P* < 0.05).

Direct presentation *in vitro* was used to ensure that all viruses expressed their antigens and present B8_20_, as described above, but for these experiments the source of primed CD8^+^ T cells was mice infected with an IAV that expresses the B8_20_ epitope as part of the neuraminidase stalk. Again as expected, all antigens were expressed, and cells infected with the minigene-B8_20_-expressing viruses were better at restimulating CD8^+^ T cells from IAV-B8_20_-immune splenocytes (Fig. 5A). At the same time, DC2.4 cells infected with viruses lacking B8 failed to restimulate the B8_20_-immune splenocytes but cells infected with all viruses were able to restimulate CD8^+^ T cells from VACV WR-infected mice. Again, this verified that our rWR and rMVA viruses were expressing antigen and presenting B8_20_ on infected cells as expected.

Next, we used heat-killed rVACV-infected cells to test the ability of B8 from these viruses to cross prime a CD8^+^ T cell response. Cells infected with wild-type WR and MVA viruses, but not the recombinants expressing minigene-B8_20_, were able to provide antigen that cross primed B8_20_-specific CD8^+^ T cells (Fig. 5B). This result for the wild-type B8 versions back up the data from Fig. 2 and 4B, again confirming that not only is the truncated B8 from MVA able to be cross primed, it is better in this assay than the full-length B8 from WR. It is possible that B8 from WR is largely lost from infected cells because it is secreted and that the truncated version from MVA is either retained or perhaps remains associated with cells to a greater extent. We also noted that the mean response to the P4 set of epitopes was lower for the minigene-B8_20_ viruses than for corresponding wild types, but this did not reach statistical significance for WR or MVA. These data together support the idea that both versions of full-length B8 are able to be cross primed but, as expected, minigene-B8_20_ is unable to cross prime CD8^+^ T cells.

Having again established that the B8_20_-presenting viruses used in this section behaved as expected in the previous experiments, we infected mice with these viruses to test them as live vaccines. When mice were infected with these viruses in the case of WR, the wild-type virus initially appeared to induce a significantly larger B8_20_-specific CD8^+^ T cell response than the minigene-B8_20_ recombinant; however, this trend was also noted for the P4 set of other VACV epitopes. To account for a possible difference in infections with these viruses, the data were normalized as a ratio of the B8_20_-specific to the total anti-VACV response. In this analysis, there was no longer any significant difference between the two WR viruses, so it seems likely that the two forms of B8_20_ were equally effective (Fig. 5C, left). In contrast, for MVA, the minigene-B8_20_-expressing virus induced a very slightly larger B8_20_-specific CD8^+^ T cell response than the wild-type virus (Fig. 5C, right). Furthermore, this difference between rMVAs remained significantly different when normalized against the total anti-VACV responses.

Thus, we concluded that for a VACV antigen expressed in its native condition there was no advantage from expression as a minigene by strain WR. However, this was not true for MVA, where the direct-priming minigene construct elicited a slightly, but significantly higher CD8^+^ T cell response. Whether this difference between strains is due to the variants of native B8 expressed by these viruses or other factors such as the extent of B8 immunodominance remains to be determined (54, 61).

### Replication of a published result for MVA expressing the OVA_257_ peptide from the delIII locus.

**FIG 6:**
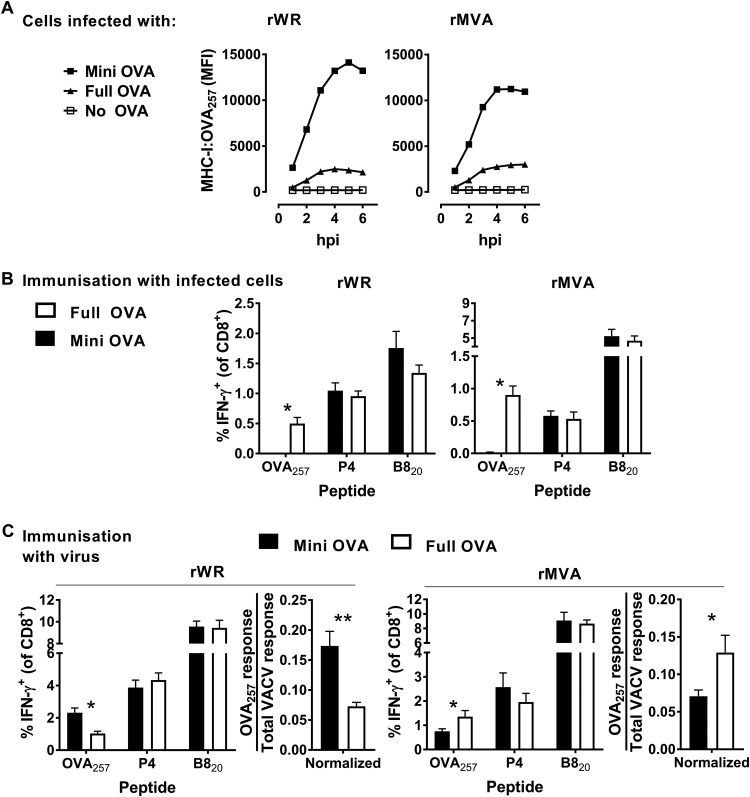
Presentation to, and priming of, CD8^+^ T cells by OVA_257_ expressed in different forms by rWR and rMVA. (A) The extent of cell surface presentation of MHC-I:OVA_257_ complexes was determined in DC2.4 cells after infection with OVA-, mini-OVA-, or no-OVA-expressing rWR and rMVA. MHC-I:OVA_257_ complexes were detected by 25D1.16 antibodies, and the MFI was determined by flow cytometry. (B) Results obtained according to the experimental design shown in Fig. 2A, except the viruses expressed versions of OVA (as shown). Epitope-specific responses are shown as the percentage of CD8^+^ T cells making IFN-γ. (C) Results obtained according to the experimental design in Fig. 3A. Mice were infected with rWR and rMVA viruses expressing versions of OVA (as shown) and, 7 days later, the epitope-specific responses were measured. The graph on the right for each VACV strain shows the OVA_257_-specific response divided by the total VACV-specific response. For panels B and C, means and standard errors of data from nine mice combined from three independent experiments are shown (*, *P* < 0.05; **, *P* < 0.01).

All the data above seemed to contradict the key study on antigen presentation from MVA by suggesting that minigenes, which require direct presentation, are an optimal form of antigen to induce a CD8^+^ T cell response using this VACV strain. So, we next used one of the same sets of rMVAs used by Gasteiger et al. (42) to repeat a published experiment, albeit using our i.d. infection route compared to the original intraperitoneal infections. These rMVAs, expressed either ovalbumin (OVA) or an minigene-OVA_257_ from the delIII locus, the site of one of the major genomic deletions in MVA. This site does not exist in the WR genome, so for comparison we used rWRs that expressed the same antigens from the TK locus, maintaining consistency with the other WR viruses used here. The presentation of OVA_257_ can be directly measured using a monoclonal antibody (25D1.16), which detects this peptide when presented by H-2K^b^, and flow cytometry (62). This is a much simpler and more strictly quantitative compared to the assays using splenocytes described for the other antigens above, so we used this method to ensure all antigens were expressed and to examine direct presentation. As has been published elsewhere, cells infected with these viruses expressing minigene-OVA_257_ presented substantially more OVA_257_ compared to those infected with rVACVs expressing full-length OVA (Fig. 6A) (42, 63).

Next, we used heat-killed virus-infected cells to test the ability of these forms of OVA to cross prime CD8^+^ T cells *in vivo*. Irrespective of virus strain, only cells infected with viruses that express full-length OVA were able to induce a CD8^+^ T cell response (Fig. 6B), which is consistent with published results for rWRs (30).

Finally, mice were infected with the OVA_257_-expressing rWR and rMVA viruses to test the optimal form of antigen in our i.d. infection model. Consistent with all the data here for other antigens, and as published previously (41), rWR encoded minigene-OVA_257_ was more immunogenic than full-length OVA (Fig. 6C, left). However, unlike our data here for other antigens and consistent with the published pattern for rMVA (42), minigene-OVA_257_ was significantly less immunogenic than the full-length protein. Again, to formally take into account possible variations in infections across these viruses, we normalized OVA_257_-specific response to the total anti-VACV response (Fig. 6C). This additional analysis supported the initial comparison made with the unnormalized values. Thus, we were able to confirm previously published results for MVA, irrespective of the different route of infection, which is consistent with route not being important for determining antigen preference for MVA (42). This result confirms a contradiction between the rMVA results obtained with OVA as published previously (42) and those acquired for all three other antigens examined here.

### Genome location and TK gene function affect the relative immunogenicity of OVA and minigene-OVA_257_ expressed by rMVAs.

To address the discrepancy noted above, we considered the differences between the OVA-expressing MVAs and other foreign gene-expressing viruses used here. These were (i) the antigen, (ii) the genomic location from which antigens were expressed, and (iii) the presence of a functional TK gene, which is inactivated when this locus is used to insert foreign antigens. We examined these differences with six new viruses: an OVA and minigene-OVA_257_ pair of rMVAs with these antigens inserted into the TK locus; a second pair of rMVAs with these antigens expressed from the intergenic space between *A11R* and *A12L*; and finally, TK^–^ variants of the rMVAs with OVA and mini-OVA_257_ expressed from the delIII region (Fig. 5A). We tested expression and direct presentation on cells infected with these pairs of rMVAs *in vitro* and the immunogenicity of OVA_257_ in mice infected with these viruses.

**FIG 7:**
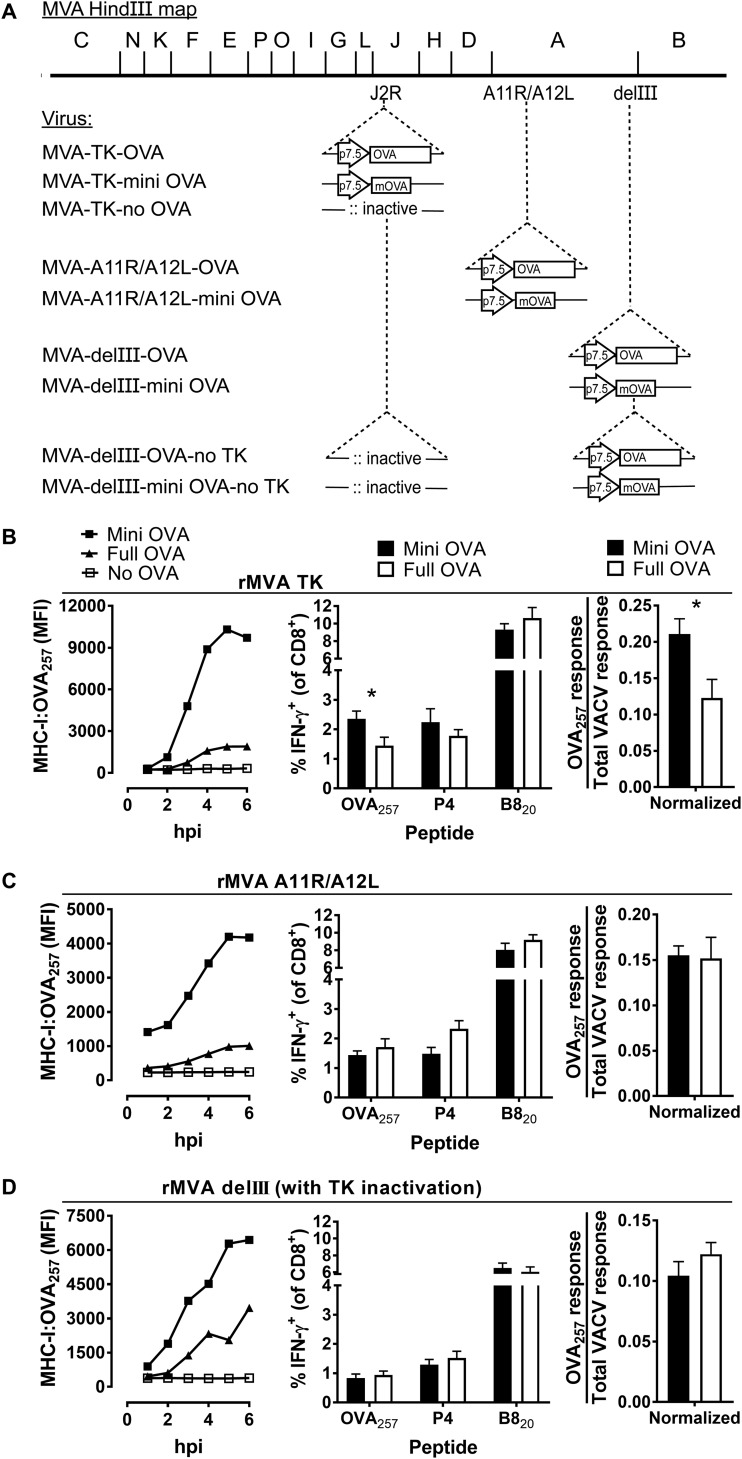
Presentation to, and priming of, CD8^+^ T cells by OVA_257_ expressed in different loci of rMVA and without a functional TK. (A) MVA genome maps showing HindIII fragments and the site of insertion of OVA antigenic constructs and the promoter (arrow). The “m” denotes the minigene (OVA_257_). (B to D, left) Extent of cell surface presentation of MHC-I:OVA_257_ complexes on DC2.4 cells infected with viruses. (B to D, middle) Mice were infected with rMVA viruses expressing versions of OVA_257_ (as shown) and, 7 days later, the responses to peptides were measured. (B to D, right) Using the data shown in the middle graphs, the OVA_257_-specific response was normalized by dividing by the total VACV-specific response. Means and standard errors of data from at least nine mice combined from three independent experiments are shown (*, *P* < 0.05).

Expression and antigen presentation on infected DC2.4 cells was tested with the 25D1.16 monoclonal antibody and flow cytometry. We found that all of the pairs of rMVAs behaved as expected with all presenting OVA_257_, and cells expressing the minigene viruses presented this epitope at higher levels than those infected with their paired full-length OVA-expressing counterpart (Fig. 7B to D, left). There were some differences in the data across the various pairs, with the *A11R/A12L* insertion and TK^–^ variant of MVA expressing OVA from the delIII locus apparently presenting OVA_257_ less efficiently than the other viruses. However, we are presenting raw mean fluorescence data and that limits direct comparisons.

When mice were infected with these viruses, two different patterns of immunogenicity were observed between full-length and minigene-OVA_257_ rMVAs. For the first pair of viruses, where antigen was expressed from the TK locus, minigene-OVA_257_ induced higher CD8^+^ T cell responses than full-length OVA (Fig. 7B, middle). This result was then tested further by normalization against the total VACV-specific responses and the difference remained in favor of minigene-OVA_257_ and was statistically significant (Fig. 7B, right). This was the opposite of the results obtained in the previous figure using rMVAs expressing OVA_257_ constructs from the delIII-based recombinants. It did, however, match the results for all the other antigen pairs that we have tested when expressed from the TK region and also for B8_20_. This suggests that the locus of expression, rather than the antigen examined (OVA), is responsible for the apparent contradiction in previous data. For the remaining pairs of rMVAs, the full-length and minigene constructs elicited an equivalent OVA_257_-specific CD8^+^ T cell response; again, this was supported by formal normalization (Fig. 7C and D, middle and right). These data suggest that genome location and TK function can contribute to the relative immunogenicity of unstable and stable polypeptides that present OVA_257_ when expressed from rMVA.

## DISCUSSION

This study started with the aim of carrying out a comprehensive side-by-side comparison of the antigen requisites and therefore priming pathway preferences for virulent VACV, strain WR, and for MVA. The results for rWR viruses were entirely in line with what was first shown more than 2 decades ago, specifically that short-lived constructs were the optimum antigen form to elicit CD8^+^ T cell responses (41). We have simply extended the data set to more antigens and expression sites in the virus and used more quantitative tools to quantify T cell responses (64). In contrast, the results for rMVAs were unexpected and so merit further discussion.

The first study of the optimum antigen forms for priming CD8^+^ T cells by rMVA examined multiple antigens and antigen forms, but all being expressed from delIII (42). These included full-length antigens, as well as an ubiquitylated version of tyrosinase, an epitope of tyrosinase that is derived from the leader sequence of that protein and the minigene-OVA_257_. There are further unpublished constructs with antigens inserted into the delIII region that all behave in the same way as shown elsewhere (I. Drexler, data not shown). Thus, there is strong evidence that whenever antigens are expressed from the delIII region of MVA, unstable antigens that can support only direct priming will induce poor CD8^+^ T cell responses compared to stable antigens. This was the current state of understanding in the field when we began our experiments. However, these published results contrast with the data for all of the pairs of rMVAs that we made for this study. We present data for several antigens and, in our case, three different genomic loci and come to the opposite conclusion. The differences across these two studies suggest strongly that the antigen form that primes optimal CD8^+^ T cell responses when expressed from rMVA differs according to the expression locus. Further support for this conclusion comes from our direct comparison of rMVAs that present the OVA_257_ epitope. We used a set of OVA-expressing rMVAs examined in the original study noted above and confirmed the published result with our model (Fig. 6C). This was followed by our own recombinants that differed only in that expression was from the TK locus and not delIII and found the opposite result (Fig. 7A). These results were supported by a rigorous normalization process to take into account any difference in infections (e.g., that might be caused by inaccurate virus titers used to infect the mice). In looking at these findings, it is pertinent to ask whether it was the immunogenicity of the full-length gene or the minigene (or both) that varied because this might suggest the priming pathway that is working with differing efficiency across the pairs. While a direct statistical comparison is not possible, across these two pairs of viruses (Fig. 6C to 7A) it can be seen that the immunogenicity of the minigene varies substantially, but the full-length construct elicits a similar response irrespective of expression locus. This is seen most clearly in the normalized data (Fig. 6C, far right, and Fig. 7A, right), where for full-length constructs the OVA_257_/total VACV-specific responses are almost identical (0.129 and 0.123), but for the minigenes they vary almost 3-fold (from 0.071 to 0.211). Taken together, the best explanation these findings, ours in the present study and those of Gasteiger et al. is that the genome location of a foreign antigen influences whether antigens designed for direct or cross priming are likely to be most immunogenic when expressed from an rMVA.

MVAs that have a transgene inserted in the delIII region differ in two ways from the majority of our viruses in which the viral TK gene was used: the genomic site of insertion and the presence of the viral TK. In teasing these two factors apart, we found that when OVA_257_ antigens were inserted in the *A11R*/*A12L* intergenic space, the full-length gene and the minigene were equally immunogenic (Fig. 7C). This result was half way between those for the corresponding rMVA with delIII and TK locus insertions. This suggested that inactivation of TK was played some role in improved immunogenicity of minigenes over full-length protein expressed from the TK locus. Further support for this was provided by our results when we inactivated TK in the original delIII-inserted rMVAs (Fig. 7D). However, we also note that when B8_20_ was expressed as a minigene from its native location in MVA, it outperformed the full protein in terms of immunogenicity (Fig. 5C). This suggests that there is a role for other factors, perhaps the antigen/epitope, e.g., if the antigen is very poorly cross primed, or the precursor frequency of T cells that recognize a particular epitope, may also be important. Taken together, however, our data establish roles both for insertion site of transgene and for TK function in determining the immunogenicity of epitope minigenes from rMVA at least for one model antigen.

The reasons why these two factors might alter priming preferences for CD8^+^ T cells are not clear. Differences in immunogenicity across sites and with variation due to TK function have been noted previously for virulent VACV, but in that case these differences were linked to transgene expression level (65). As noted above, in every experiment we found enhanced presentation of epitopes from minigenes over those from full-length proteins on infected DC2.4 cells. In addition, cells infected with MVAs that express full-length OVA generate enough of this protein to cross prime CD8^+^ T cell responses *in vivo* as shown in Fig. 6B for the delIII insertion and as published previously for a TK insertion (37). Having said this, we are unable to know how much of each full-length antigen is expressed and more importantly its capacity to cross prime *in vivo*. However, differences in immunogenicity not linked to expression have been seen previously for MVA (66). We speculate that differences in genomic insertion sites can change the interaction of rMVA with the DCs responsible for priming CD8^+^ T cells. In the case of rMVAs that used the delIII region, while no functional genes are disrupted, we wonder whether the transcription that is driven into the neighboring viral genes alters their expression, leading to changes in infected DCs. In support of this general concept, we have noticed that insertions that include an expressed gene into the *A11R*/*A12L* intergenic space can alter the immunogenicity of multiple native VACV epitopes, whereas a promoterless insertion did not have this effect (L. C. W. Lin and D. C. Tscharke, unpublished data). In contrast to these loci, use of the TK region leads to a loss of TK function. Indeed, this locus was originally chosen because this phenotype provided a selectable marker for recombinant viruses (67–69). VACV TK functions to phosphorylate thymidine, producing dTMP, which after a series of phosphorylation events is utilized in *de novo* DNA synthesis (70). *In vitro*, the function of this gene is not required, but the loss of TK is substantially attenuating for virulent VACVs *in vivo*, reducing viral loads (54, 71). However, MVA fails to replicate *in vivo*, and so we did not expect to find any impact of TK deletion. Further, TK deletion from WR does not substantially change the specificity of CD8^+^ T cell responses, despite the attenuation noted above (54). Finally, although MVA does replicate its genome in some cells, and so inhibition of this DNA synthesis might have an impact, in the case of DCs MVA aborts infection at an earlier stage, suggesting that this is not relevant for direct priming (72). It remains possible that there is some effect of TK function on DCs, perhaps prolonging their lifespan during infection. We have explored this *in vitro* and found no impact of TK expression from MVA on DC viability (Y. C. Wong and D. C. Tscharke, unpublished data), but our *in vitro* DC cultures are unlikely to model the situation *in vivo* very faithfully, all the more so given that a second wave of presentation has been shown to be required for full immunogenicity of VACVs (43). The role of TK and of transcription that runs into neighboring genes in MVA vaccines requires a more thorough investigation.

Our data may also have implications for our understanding of the ability of MVA to support direct priming in general. While there is one well-characterized exception, namely, minigene-IAV PA_224_, minigenes (either ER targeted or cytosolic) and other rapidly degraded constructs have never been found to be cross presented. This is consistent over multiple studies, and the expression of these antigens can therefore be used to establish the effectiveness of direct presentation (30, 38, 42, 51, 52, 56, 73, 74). Thus, the finding by Gasteiger et al. that multiple rapidly degraded proteins were poorly immunogenic suggested that direct priming is inefficient for MVA in general. We now find here that several rMVA minigene viruses are able to elicit CD8^+^ T cell responses at least as effectively as full-length proteins and often significantly better. This includes four antigens expressed from four loci in the virus, including under a natural promoter in the native location. The only exception is minigene-OVA_257_ expressed from delIII. The weight of evidence then suggests that MVA in general supports efficient direct priming of CD8^+^ T cells. There is likely to be a contribution by cross priming as well, but we are unable to determine the relative importance of these pathways for nonrecombinant MVA.

From a practical perspective, our data show that MVA can be an efficient vector for the delivery of antigens for direct presentation. Having noted this general rule, it is clear that the use of the delIII region, and perhaps others yet to be determined, to make rMVAs creates exceptions. This remains important because delIII was the original and remains a commonly used site for the introduction of genes encoding foreign antigens in rMVAs (14, 75). It is also necessary for the interpretation of any experiments where rMVA are used to dissect mechanisms of CD8^+^ T cell priming. Indeed, the finding that the XCR1^+^ DCs required for a fully functional CD8^+^ T cell response used exclusively cross priming was made using H-2K^bm1^ mice infected with an rMVA that expressed H-2K^b^ and OVA from the delIII region. Finally, we have only examined systemic responses and, for the priming of resident memory populations (Trm), cross priming has been shown to be important using mouse knockout models (76).

In conclusion, we show that directly priming minigenes are optimal for CD8^+^ T cell priming by virulent (WR) and attenuated (MVA) rVACV, with the notable exception of rMVAs that express antigens from the DelIII region. Minigenes, while they are not likely to be used as vaccines themselves, are a model for all forms of rapidly degraded antigens. Other forms, such as ubiquitin fusions or polyepitope constructs, are more practical antigens for vaccines because they can induce responses to multiple epitopes in the context of multiple MHC allomorphs. MVAs with insertions into delIII are common, but it is not the only site used. Indeed, we are not the only group to use rMVAs with antigens expressed from the TK locus, and some of these vaccines have advanced to clinical trials, so our findings have practical implications (22, 77–83). The findings presented here in general highlight that there remain many wrinkles to iron out in our understanding of antigen presentation, even for well-studied viral vectors that have been used in human clinical trials.

## MATERIALS AND METHODS

### Mice.

Specific-pathogen-free, female, 7- to 14-week-old C57BL/6 mice were obtained from the Australian Phenomics Facility (Canberra, Australia) or ARC (Perth, Australia). All experiments were conducted according to relevant ethical requirements that were approved by the Australian National University Animal Ethics and Experimentation Committee (protocols F.BMB.38.08, A2011.001, A2013.037, and A2016.045).

### Cells and nonrecombinant viruses.

For cross presentation assays, 293A cells (ATCC, CRL-1573) were used as antigen donor cells. The C57BL/6 mouse-derived, dendritic cell-like cell line DC2.4 was used for *in vitro* presentation assays (84). Unmodified Western Reserve vaccinia virus (VACV WR, ATCC VR1354) and MVA were originally a gift from B. Moss (National Institutes of Health, Bethesda, MD). Influenza A virus (IAV) strain PR8 was provided by C. Goodnow (ANU, Canberra Australia). HSV strain KOS was provided by F. Carbone (University of Melbourne, Melbourne, Australia). All strains were grown and titrated according to standard methods.

### Recombinant viruses and virus construction.

The VACVs used here are listed in Table 1 with descriptions and origins, if not made for this study. With the exception of the minigene-B8_20_ WR and MVA, which used the native B8 promoter, all antigens were expressed from the VACV p7.5 promoter. Homologous recombination between appropriately designed transfer plasmids supplied by transfection and VACV genomes provided by infection was used to make the new viruses required. Transfer plasmids were based on pSC11 and p7.5GB-ins for insertions into the TK and *A11R*/*A12L* regions, respectively (69, 85). Briefly BHK-21 or 293A cells were infected with MVA or WR, respectively, at a multiplicity of infection (MOI) of 0.05 in Dulbecco modified Eagle medium (DMEM) supplemented with 2% fetal bovine serum (FBS) and incubated at 37°C and 5% CO_2_ for 1 h. The inoculum was then removed, and the transfer plasmid was added in a preincubated transfection mix of plasmid, Lipofectamine 2000 (Life Technologies), and DMEM. Infected cell cultures were incubated with transfection mix for 2 days at 37°C and 5% CO_2_ before the virus was released by repeated freeze-thaw cycles and sonication. Recombinant viruses were isolated via serial-step-dilution plaque purification and transient dominant selection using blasticidin/green fluorescent protein (GFP) expression or direct staining for β-galactosidase (pSC11-based plasmids only) as previously described (85). PCR was used to identify recombinant viruses, and the recombinant region was verified by sequencing. The IAV strain PR8 that included B8_20_ in the stalk of the neuraminidase protein was generated using a plasmid-based reverse genetic system as previously described for a virus that included OVA_257_ in the same way (86, 87).

**TABLE 1:** Viruses used in this study

Virus^*a*^	Recombinant details	Source or reference
MVA	Wild-type VACV strain MVA	89
WR	Wild-type VACV strain WR	90
WR-Full-gB	Full-length HSV gB in TK	51
WR-ESmini-gB	ER-targeted minigene-gB_498_ in TK	50
MVA-Full-gB	Full-length HSV gB in TK	This study
MVA-ESmini-gB	ER-targeted minigene-gB_498_ in TK	This study
WR-TK-	Insertional inactivation of TK	69
MVA-TK^-^	Insertional inactivation of TK	This study
WR-Full-PB1-F2	Full-length IAV PB1F2 in TK	58
WR-ESmini-PB1-F2	Minigene-IAV PB1F2_62_ in TK	53
MVA-Full-PB1-F2	Full-length IAV PB1F2 in TK	This study
MVA-ESmini-PB1-F2	Minigene-IAV PB1F2_62_ in TK	This study
IAV-miniB8	IAV PR8 with sequence encoding B8_20_ inserted into the neuraminidase stalk	This study
WR-delB8	*B8R* deleted	60
WR-delB8-miniB8	*B8R* replaced with minigene-B8_20_	85
MVA-delB8	*B8R* deleted	91
MVA-delB8-miniB8	*B8R* replaced with minigene-B8_20_	This study
WR-TK-OVA	Full-length OVA in TK	41
WR-TK-miniOVA	Minigene-OVA_257_ in TK	92
MVA-delIII-OVA	Full length OVA in delIII	42
MVA-delIII-SIINFEKL	Minigene-OVA_257_ in delIII	42
MVA-TK-OVA	Full-length OVA in TK	This study
MVA-TK-miniOVA	Minigene-OVA_257_ in TK	37
MVA-A11R/A12L-OVA	Full-length OVA in *A11R*/*A12L*	This study
MVA-A11/A12-SIINFEKL	Minigene-OVA_257_ in *A11R*/*A12L*	This study
MVA-delIII-OVA-delTK	Full-length OVA in delIII with insertional inactivation of TK	This study
MVA-delIII-SIINFEKL-delTK	Minigene-OVA_257_ in delIII with insertional inactivation of TK	This study

a The original nonrecombinant parent virus is indicated by the first letters of each name: WR, MVA, or IAV. The order of the viruses is as they appear in the main text, and they are separated into groups according to the figures and legends in which they are described.

### Peptides.

Synthetic peptides made to match the sequences of the epitopes of interest (Table 2) were purchased from GenScript (Piscataway, NJ) or Mimotopes (Clayton, Victoria, Australia). Peptide master stocks were made in 100% dimethyl sulfoxide (DMSO), generally at 1 × 10^−2^ M, and then diluted in serum-free DMSO for use in assays at a final concentration of 1 × 10^−7^ M.

**TABLE 2:** Synthetic peptides used in this study

Peptide^*a*^	Origin^*b*^	Sequence	MHC	Reference(s)
A3_270_	VACV, A3_270–277_	KSYNYMLL	H-2K^*b*^	33
A47_138_	VACV, A47_138–146_	AAFEFINSL	H-2K^*b*^	61
A47_171_	VACV, A47_171–180_^*c*^	YAHINALEYI	H-2K^*b*^	55
L2_53_	VACV, L2_53–61_	VIYIFTVRL	H-2K^*b*^	33
B8_20_	VACV, B8_20–27_	TSYKFESV	H-2K^*b*^	61
gB_498_	HSV-1 gB_498–505_	SSIEFARL	H-2K^*b*^	93
OVA_257_	Chicken, OVA_257–264_	SIINFEKL	H-2K^*b*^	94, 95
PB1F2_62_	IAV strain PR8, PB1F2_62–70_	LSLRNPILV	H-2D^*b*^	58

a Data from the first four peptides (A3_270_, A47_138_, A47_171_, and L2_53_) were pooled and are referred to as P4.

b That is, the virus/organism, protein, and position of the peptide (in subscript amino acid numbers) in protein.

c Numbering from WR, the corresponding peptide from MVA is A47_157–166_.

### *In vitro* assay for presentation of MHC-I–peptide complexes on infected cells.

For epitopes other than OVA_257_, DC2.4 cells were infected with VACVs for 2, 4, or 6 h before being placed on ice so that presentation at all time points could be tested simultaneously. Presentation was tested by coculturing the infected DC2.4 with splenocytes from an appropriately immunized mouse at a stimulator/effector ratio of 1:5 for a total of 4 h at 37°C with 5% CO_2_ and adding brefeldin A to 50 μg/ml after the first hour. Cells were then labeled for CD8 (anti-mouse CD8α-PE; BioLegend, clone 53-6.7) and intracellular IFN-γ (anti-mouse IFN-γ-APC; BioLegend, clone XMG1.2) staining for flow cytometric analysis (88). Events were gated on sequentially on SSC × FSC, SSC × CD8, and CD8 × IFN-γ plots to determine the percentages of CD8^+^ cells that were IFN-γ^+^. The same splenocytes were stimulated with synthetic peptide with a sequence matching the epitope of interest, and the conditions for incubation and staining were as described above. The fraction of CD8^+^ T cells making IFN-γ in these peptide-stimulated cultures was set at 100%, and results from cocultures with infected cells are presented relative to this maximum response. To determine presentation levels of H-2K^b^-OVA_257_, DC2.4 cells were infected for the times noted before labeling with 25D1.16-APC antibody (BioLegend). Labeled cells were fixed in 1% paraformaldehyde before flow cytometric analysis. Events were gated on a SSC × FSC plot, and the mean fluorescence intensity (MFI) of the population was determined.

### Infection of cells and immunization of mice to determine cross presentation *in vivo*.

Single cell suspensions of 293A cells were incubated with indicated viruses at an MOI of 5 PFU/cell in DMEM for 1 h at 37°C with shaking (200 rpm). Infected cells were resuspended in DMEM supplemented with 2% FBS, and infection continued for 5 h at 37°C with slow rotation to stop cells settling which were then counted. To inactivate the residual virus inoculum and inhibit viral replication, infected cells were resuspended in phosphate-buffered saline (PBS) and heat treated at 60°C for 60 min prior to i.d. immunization. Mice were anesthetized with isoflurane and immunized with 2 × 10^6^ cells in 10 μl by i.d. injection of the left ear pinnae. Seven days later, the mice were euthanized, and spleens were taken to determine the extent of the responses to relevant epitopes using stimulation *in vitro* with synthetic peptides and staining for CD8 and IFN-γ, as described above.

### Infection of mice with viruses.

Viruses were diluted in PBS, and 2 × 10^6^ PFU was injected i.d. in the left ear pinnae. After 7 days, the mice were euthanized, and spleens were taken to determine the extent of the CD8^+^ T cell responses to various epitopes by stimulation with synthetic peptides and staining for CD8 and IFN-γ, as described above.

### Flow cytometry and statistical analysis.

Flow cytometry data acquisition was done using an LSR-II flow cytometer (BD Biosciences). Data were analyzed as described above with FlowJo 8.8.4 software (Tree Star, Ashland, OR). Statistical analysis was performed with GraphPad Prism 7 Software. Differences between means were tested using a two-way *t* test and were considered significant when the *P* value was <0.05.
